# Cholesterol-Induced Non-Alcoholic Fatty Liver Disease and Atherosclerosis Aggravated by Systemic Inflammation

**DOI:** 10.1371/journal.pone.0097841

**Published:** 2014-06-05

**Authors:** Eung Ju Kim, Baek-hui Kim, Hong Seog Seo, Yong Jik Lee, Hyun Hee Kim, Hyun-Hwa Son, Man Ho Choi

**Affiliations:** 1 Cardiovascular Center, Division of Cardiology, Department of Internal Medicine, Korea University Guro Hospital, Korea University College of Medicine, Seoul, Korea; 2 Department of Pathology, Korea University Guro Hospital, Korea University College of Medicine, Seoul, Korea; 3 The Korea University-Korea Institute of Science and Technology (KU-KIST) Graduate School of Converging Science and Technology, Korea Institute of Science and Technology, Seoul, Korea; 4 Future Convergence Research Division, Korea Institute of Science and Technology, Seoul, Korea; University Heart Center Freiburg, Germany

## Abstract

Although triglyceride accumulation in the liver causes non-alcoholic fatty liver disease (NAFLD), hypercholesterolemia is also a main cause of NAFLD as well as atherosclerosis. However, NAFLD and atherosclerosis have not been investigated simultaneously in animal models fed a high-cholesterol diet. Moreover, it is unclear whether systemic inflammation can exacerbate both pathologies in the same model. Accordingly, this study investigated the effect of additional systemic inflammation on NAFLD and atherosclerosis induced by cholesterol overload in wild animals. New Zealand white rabbits were divided into 4 groups: groups I (control) and II received normal chow, and groups III and IV received a 1% cholesterol diet. To induce inflammation via toll-like receptor (TLR)-4 signaling, groups II and IV received subcutaneous injections of 0.5 mL of 1% carrageenan every 3 weeks. After 3 months, total cholesterol markedly increased in groups III and IV, and the serum expressions of systemic inflammatory markers were elevated in the groups II–IV. Early NAFLD lesions (e.g., mild fatty changes in the liver with sporadic fibrosis) and atherosclerosis (e.g., intimal hyperplasia composed of foam cells) were observed in both the liver and aorta specimens from group III, and advanced lesions were observed in group IV. The expressions of inflammatory cellular receptors, TLR-2 and TLR-4, in the aorta gradually increased from group I to IV but were similar in the liver in groups II–IV. Cholesteryl ester (CE) levels were higher in group IV than in group III, although the difference was not significant. CE levels in the aorta were similar between groups III and IV. Systemic inflammation can simultaneously exacerbate existing early lesions due to cholesterol overload in both the liver and aorta of rabbits. However, the cellular response of inflammatory receptors and expression of cholesterol metabolites differ between these organs.

## Introduction

Non-alcoholic fatty liver disease (NAFLD) is common in the general population, and it occurs even more frequently in patients with metabolic syndrome [1], [2]. Patients with NAFLD have an increased risk of cardiovascular disease (CVD) [2], [3] because these diseases share several risk factors and surrogate markers [3], [4]. In addition, NAFLD is often associated with atherosclerotic signs including the presence of carotid plaques [5] and coronary arterial calcium [6].

The pathogenesis of NAFLD is based on a “two-hit hypothesis” [7], [8]. The first hit involves triglyceride accumulation within hepatocytes, which results in simple steatosis; this is mainly attributed to insulin resistance associated with obesity [9], and it increases the vulnerability of the liver to further injury [10]. The second hit is primary lipotoxicity caused by oxidative stress from increased lipid peroxidation, high reactive oxygen species production within hepatocytes, mitochondrial dysfunction, and inflammation. Progression to steatohepatitis and fibrosis through the activation of hepatic stellate cells may subsequently occur in response to these biological processes [11]. Many of the biological substrates and conditions involved in the development of NAFLD, including insulin resistance and proinflammatory cytokines, exert the same effects on arteries, resulting in atherosclerosis. This may help explain the association between NAFLD and CVD. However, although triglycerides accumulate in hepatocytes, they do not accumulate in the arterial wall.

Recent studies investigated NAFLD resulting from hypercholesterolemia [12]–[15]. Because cholesterol can accumulate in both arteries and the liver, NAFLD caused by cholesterol overload may be accompanied by atherosclerosis in the arteries. However, it is unclear whether cholesterol accumulation in the liver results in simple first-hit steatosis or whether second-hit inflammation facilitates the progression to steatohepatitis through inflammatory cell infiltration and fibrosis. Therefore, it is unknown if cholesterol accumulation primes the liver for further injury as is observed in cases of triglyceride-induced fatty liver.

Inflammation can play an important role in atherosclerosis progression. Moreover, in NAFLD, steatohepatitis is an important factor that suggests progression to advanced liver disease. However, it is unclear if systemic inflammation affects both atherosclerosis and NAFLD induced by hypercholesterolemia. Therefore, this study investigated whether additional remote inflammation can simultaneously exacerbate underlying atherosclerosis and NAFLD induced by cholesterol overload.

## Materials and Methods

### Animal preparation

Sixteen male New Zealand white rabbits (12 weeks of age) were maintained under standardized conditions (21°C, 41–62% humidity) with a regular day/night (10/14 hours) cycle and free access to water and a laboratory diet. All animal experiments complied with the Korea University Animal Science Rules and Regulations, and the protocols were approved by the Korea University Institutional Animal Care and Use Committee (approval number: KUIACUC20110627-1). Furthermore, the experimental procedures and housing conditions were approved by the Committee of Animal Experimentation, Hiroshima University. The animals were randomly assigned to 1 of 4 groups as follows: group I, control; group II, subcutaneous carrageenan injection; group III, cholesterol diet; and group IV, subcutaneous carrageenan injection and cholesterol diet. Groups I and II received standard maintenance chow diets (K-H4 pellets, Sniff), while groups III and IV received the same diet supplemented with 1.0% (weight/weight) cholesterol. To induce inflammation via toll-like receptor (TLR)-4 signaling, groups II and IV received subcutaneous injections of 0.5 mL of 1% carrageenan every 3 weeks. After 3 months of treatment, all animals were killed. Blood samples were obtained from the inferior vena cava, and the animals were subsequently perfused with phosphate-buffered saline (PBS) followed by formalin-sucrose (4% paraformaldehyde and 5% sucrose in PBS [pH 7.4]). The liver and entire arterial system from the proximal ascending aorta to the bifurcation of the iliac artery were dissected and placed in 10% formalin for immersion fixation.

### Blood biochemical and inflammatory marker measurements

Blood samples for complete blood counts were collected in tubes with EDTA and analyzed by using Sysmex XE-2100 (Kobe, Japan). Commercially available assay kits were used for biochemical measurements. Enzymatic calorimetry (Roche Diagnostics GmbH; Mannheim, Germany) was used to measure the concentrations of total cholesterol, high-density lipoprotein cholesterol (HDL-C), low-density lipoprotein cholesterol (LDL-C), and triglycerides. C-reactive protein (CRP) was measured by using an immunonephelometric assay (BN ProSpec System protein analyzer, Siemens Diagnostic Healthcare Inc., Deerfield, IL, USA). Serum assays for inflammatory markers were conducted by using a 4-plex cytokine Milliplex panel (Millipore Corporation, Billerica, MA, USA) as recommended for cytokines interleukin (IL)-1β, IL-6, and tumor necrosis factor (TNF)-α. Acquisition was performed on a Luminex 100 platform, and the analysis was carried out by using Multiplex analyst (Millipore Corporation). Alanine aminotransferase (ALT), aspartate aminotransferase (AST), and albumin were measured to evaluate liver function.

### Real-time PCR for TLR-2 and TLR-4 mRNA expression

Total RNA was extracted from tissues and purified by using an RNeasy kit (Qiagen, Venlo, Netherlands). RNA quality was assessed by using a NanoDrop 1000 (Thermo Fisher, Waltham, MA, USA). cDNA was synthesized by using an iScript synthesis kit (Bio-Rad, Hercules, CA, USA). Real-time quantitative reverse-transcription polymerase chain reaction (qRT-PCR) was performed on an ABI Prism 7300 (Applied Biosystems, Foster City, CA, USA) by using iTaq SYBR Green Supermix with ROX (Bio-Rad). Non-template controls were incorporated into each PCR run. Specific mRNA levels of all genes of interest, including TLR-2 and TLR-4, were normalized to that of a housekeeping gene (*gapdh*) and expressed as changes normalized to controls. Primers and probes from TaqMan were used for gene expression analysis with qRT-PCR (assay IDs: Oc03824728s1 for TLR-2 probe and Oc03398502m1for TLR-4).

### Quantification of the mRNA expressions of inflammatory genes in the aorta and liver

Total RNA was extracted by using TRIzol reagent according to the manufacturer's instructions. Complementary DNA was synthesized from total RNA by using the Power cDNA Synthesis Kit, and PCR for IL-1β, macrophage chemoattractant protein (MCP)-1, and TNF-α, and GAPDH were performed by using a PCR Premix kit. Primer sequences are shown in Table S1 in File S1.

### Pathologic evaluation of the liver and aorta

Macroscopic and microscopic analyses of specimens were performed in a blinded fashion. Sections were embedded in paraffin, and stained with hematoxylin & eosin and Sirius red by using standard protocols. The livers were scored according to the NAFLD activity score system by 2 pathologists blinded to the treatment groups [16]. Early atherosclerotic lesions were defined as lesions with intimal hyperplasia composed of foam cells in the aorta, and advanced lesions were defined as lesions with a necrotic core and/or vascular calcification and/or medial layer interruption. In NAFLD, less-advanced lesions were defined as early lesions in terms of fibrotic change among the groups.

### Measurement of cholesterols in serum, the liver, and the aorta

The concentrations of free cholesterol, cholesteryl esters (CEs), and hydroxycholesterols (OHCs) were quantified as described previously [17], [18].

#### Chemicals

Cholesterol, CEs, and OHCs were purchased from Sigma (St. Louis, MO, USA). Deuterium-labeled internal standards, 2,2,3,4,4,6-*d*_6_-cholesterol, 2,2,3,4,4,6-*d*_6_-cholesteryl stearate, 25,26,26,26,27,27,27-*d*_7_-4β-hydroxycholesterol, and 25,26,26,26,27,27,27-*d*_7_-27-hydroxycholesterol were obtained from C/D/N Isotopes (Pointe-Claire, Quebec, Canada) and Avanti Polar Lipids (Alabaster, AL, USA). The trimethylsilylation (TMS) reagent, *N*-methyl-*N*-trimethylsilyltrifluoroacetamide (MSTFA), ammonium iodide (NH_4_I), and dithioerythritol (DTE) were acquired from Sigma. The hybrid solid-phase extraction (SPE)-precipitation cartridge (H-PPT, 1 mL, 30 mg) was supplied by Supelco (Bellefonte, PA, USA). All organic solvents were analytical, high-performance liquid chromatography-grade chemicals, and were purchased from Burdick & Jackson (Muskegon, MI, USA).

#### Instrument conditions

Gas chromatography-mass spectrometry (GC-MS) was performed using an Agilent 6890 Plus gas chromatograph interfaced with a single-quadrupole Agilent 5975C MSD (Agilent Technologies, Palo Alto, CA, USA); the electron energy was 70 eV, and the ion source temperature was 230°C. Each sample (2 µL) was injected in split mode (10∶1) at 280°C and separated through a MXT-1 cross-linked dimethylpolysiloxane capillary column (30 m×0.25 mm I.D., 0.25 µm film thickness, Silcosteel-treated stainless steel; Agilent Technologies). The oven temperature was initially set at 260°C for 3 min, increased to 320°C by increasing the temperature at a rate of 10°C/min, increased to 330°C at a rate of 2°C/min and held for 8 min, and finally increased to 380°C at30°C/min rate and held for 3 min. The carrier gas was ultra-high-purity helium at a column head pressure of 75.8 kPa (column flow: 1.1 mL/min, oven temperature: 260°C). For quantitative analysis, the characteristic ions of each compound were determined as their TMS derivatives. Peaks were identified by comparing the retention time and matching the height ratios of characteristic ions.

#### Pre-treatment of serum samples

Serum samples (20 µL) were spiked with 20 µL of internal standard (IS) mixtures (i.e., *d*_6_-cholesterol and *d*_6_-cholesteryl stearate, 100 µg/mL; *d*_7_-4β-hydroxycholesterol and *d*_7_-27-hydroxycholesterol, 20 µg/mL) and added to 0.5 mL of methanol. The mixtures were vortexed for 5 min and centrifuged for 2 min at 12,000 rpm for protein precipitation. The samples were loaded into H-PPT cartridges and eluted 3 times with 0.5 mL of methanol. The combined methanol eluates were evaporated using an N_2_ evaporator at 40°C and dried in a vacuum desiccator over P_2_O_5_/KOH for at least 30 min. Finally, the dried residues were derivatized in 40 µL of MSTFA/NH_4_I/DTE (500∶4∶2, v/w/w) for 20 min at 60°C, and 2 µL of the resultant mixture was injected for GC-MS analysis under the selected-ion monitoring (SIM) mode.

#### Pre-treatment of tissue samples

Tissue samples (20 mg) from the liver and aorta were homogenized using a TissueRuptor (Qiagen Inc., Valencia, CA, USA) in 2 mL of 0.2 M acetate buffer (pH 5.2) for 1 min. The IS mixture (20 µL) was added to the tissue extract, and the sample was extracted twice using 2.5 mL of ethyl acetate/*n*-hexane (2∶3, v/v). The organic solvent was evaporated using an N_2_ evaporator at 40°C, 0.5 mL of methanol was added to the sample, and the sample was vortexed for 30 s. The mixture was loaded into a Hybrid SPE precipitation cartridge and eluted using 0.5 mL of methanol. The eluate was evaporated and dried in a vacuum desiccator over P_2_O_5_/KOH for at least 30 min. The dried residue was derivatized in 40 µL of MSTFA/NH_4_I/DTE (500∶4∶2, v/w/w) for 20 min at 60°C, and 2 µL of the resultant mixture was used for GC-SIM/MS analysis.

### Lipoprotein electrophoresis of serum

#### Agarose gel electrophoresis

Plasma and the isolated lipoproteins were subjected to electrophoresis on 1% agarose gels by using the Corning Universal Electrophoresis system (Corning, New York, NY, USA). The gels were stained with Fat Red 7B and Coomassie blue for lipids and proteins, respectively, as described previously [19]. The Fredrickson-Levy-Lees classification of hyperlipemia is based on the differentiation of lipoproteins according to their mobility in zone electrophoresis [20].

### Statistical analysis

Continuous variables are expressed as mean ± standard deviation (SD). Overall differences in variables among the 4 groups were analyzed using the Kruskal–Wallis test. Differences between 2 groups were evaluated using the Mann–Whitney *U*-test. Because the distribution of CRP was highly skewed to the left, the data were log-transformed, and the correlation with inflammatory cytokines was analyzed by calculating Spearman's rank correlation coefficients. The level of statistical significance was set at *P*<0.05. All statistical analyses were performed using SPSS (version 20.0; SPSS Inc., Chicago, IL, USA) and Sigmaplot 10.0 software (SYSTAT Software Inc., San Jose, CA, USA).

## Results

### Biochemical parameters and inflammatory marker levels in peripheral blood

The serum levels of total cholesterol, LDL-C, and HDL-C were markedly elevated in the groups that received the cholesterol diet (i.e., groups III and IV). Total cholesterol, LDL-C, and HDL-C levels tended to be lower in group IV than in group III, although the differences were not statistically significant (**Table 1**). AST levels were higher in the rabbits fed a high-cholesterol diet (i.e., groups III and IV) than those fed the control chow (i.e., groups I and II). Meanwhile, ALT levels did not differ significantly among the groups. Serum albumin level, a surrogate marker of hepatic synthetic ability, tended to be lower in groups III and IV than in groups I and II, but the differences were not significant.

**Table 1:** Levels of serum lipids and biochemical parameters in the 4 groups.

	Group I	Group II	Group III	Group IV	*p-value*
TC(mg/dL)	23.4±4.6^a^	29.0±13.8^a^	2359.2±1062.8^b^	1911.2±521.8^b^	0.002
TG(mg/dL)	42.8±30.5^a^	52.4±26.9^a^	163.0±253.7^b^	202.8±220.1^b^	0.429
HDL-C(mg/dL)	14.8±5.0^a^	19.2±9.0^a^	583.8±300.1^b^	322.6±148.2^b^	0.002
LDL-C(mg/dL)	4.4±3.2^a^	6.8±4.7^a^	2268.0±1015.2^b^	1830.4±512.6^b^	0.002
Albumin(mg/dL)	3.48±0.19^a^	3.43±0.2^ a^	3.22±0.11^a^	3.20±0.20^a^	0.068
ALT(IU/L)	45.6±16.8^a^	40.5±16.1^a^	46.2±14.7^a^	50.8±21.7^a^	0.86
AST(IU/L)	19.8±5.1^a^	18.0±6.4^a^	37.0±11.0^b^	29.6±9.1^b^	0.017

*p*-values represent overall differences among groups as determined by the Kruskal–Wallis test.

a,b The same letters indicate no statistical significance according to the Mann–Whitney *U*-test.

CGN, carrageenan; CHO, cholesterol; TC, total cholesterol; TG, triglyceride; HDL-C, high-density lipoprotein cholesterol; LDL-C, low-density lipoprotein cholesterol; ALT, alanine aminotransferase; AST, aspartate aminotransferase.

Levels CRP and proinflammatory cytokines including IL-1, IL-6, and TNF-α were significantly higher in groups III and IV than in groups I and II (**Table 2**). White blood cell (WBC) count tended to be higher in groups III and IV than in groups I and II, although the differences were not significant (**Table 2**); in particular, WBC count tended to be higher in group III than in group IV. As expected, serum levels of inflammatory markers were the highest in group IV but were not significantly different from those of group III. The serum levels of proinflammatory cytokines were closely correlated with each other and with the overall level of inflammatory markers (**Table 3**). Higher CRP levels were correlated with higher levels of proinflammatory cytokines.

**Table 2:** Levels of serum inflammatory cytokines and reactants, and white blood cell count in peripheral blood.

	Group I	Group II	Group III	Group IV	*p-value*
IL-1(pg/mL)	10.8±1.0^a^	15.2±3.8^b^	24.1±8.1^c^	32.3±15.4^c^	0.001
IL-6(pg/mL)	11.8±5.1^a^	32.3±15.4^a,b^	69.6±15.6^b^	63.5±23.5^b^	0.016
TNF-α(pg/mL)	59.0±40.9^a^	104.6±46.5^a,b^	159.7±34.1^b,c^	209.5±61.2^c^	0.004
CRP(mg/L)	5.5±3.4^a^	23.3±13.8^b^	87.9±74.7^c^	102.0±111.0^c^	0.002
WBC(x10^3^/mm^3^)	9.96±5.53^a^	10.30±5.44^a^	14.02±4.65^a^	12.69±2.62^a^	0.214
Neutrophils(%)	29.6±4.4	32.0±9.6	39.8±13.5	41.7±13.3	0.25
Monocytes(%)	0.9±0.9	0.7±0.5	1.4±0.5	0.9±0.6	0.41
Lymphocytes(%)	59.6±6.0	58.4±12.5	48.1±13.6	49.4±15.3	0.401

*p*-values represent overall differences among groups as determined by the Kruskal–Wallis test.

a–c The same letters indicate no statistical significance according to the Mann–Whitney *U*-test.

CGN, carrageenan; CHO, cholesterol; IL, interleukin; TNF, tumor necrosis factor; CRP, C-reactive protein, WBC, white blood cells.

**Table 3:** Correlations among log CRP and cytokines.

	Log CRP	IL-1	IL-6
IL-1	0.729 (<0.001)		
IL-6	0.543 (0.011)	0.770 (<0.001)	
TNF-α	0.699 (<0.001)	0.781 (<0.001)	0.692 (0.001)

Data are Spearman's correlation coefficients, *r*, with (*P*-values).

CRP, C-reactive protein; IL, interleukin; TNF, tumor necrosis factor.

### mRNA expressions of TLR-2, TLR-4, and other inflammatory markers in the aorta and liver

**Figure 1:**
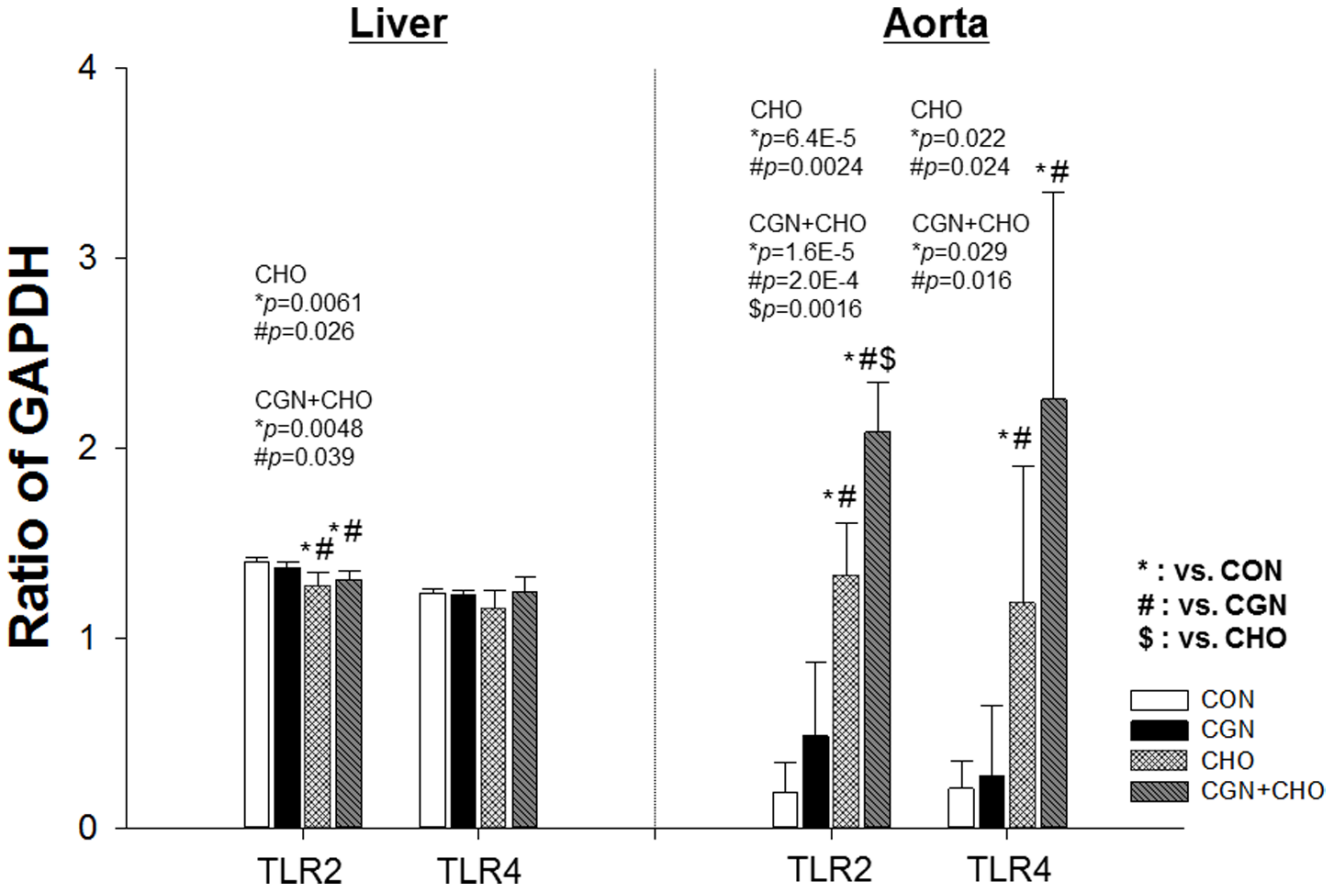
Hepatic and aortic mRNA expression of toll-like receptor (TLR)-2 and TLR-4. The baseline mRNA expressions of TLR-2 and TLR-4 were higher in the liver than in the aorta. Changes in mRNA expression in response to the cholesterol diet and/or carrageenan injection were substantially different between tissues. mRNA expressions of TLR-2 and TLR-4 in the aorta but not the liver were markedly different among the 4 groups. CON: control; CGN: carrageenan injection; CHO: cholesterol diet; CGN+CHO: carrageenan injection and cholesterol diet. Bars indicate 1 standard deviation.

Basal TLR-2 and TLR-4 mRNA expression levels were significantly higher in the liver than in the aorta, and the responses to the cholesterol diet and/or carrageenan injection into subcutaneous tissue were differed substantially between the 2 tissues. In the liver, the expressions of these receptors were slightly elevated in rabbits that received the cholesterol diet and/or carrageenan injection (**Fig. 1**), although the differences were not significant. In contrast, the mRNA expressions of TLR-2 and TLR-4 in the aorta were markedly different among the 4 groups. These results indicate the expressions of these TLRs induced systemic inflammation, and they were upregulated because of the cholesterol diet (**Fig. 1**). In the liver, there were no differences in the mRNA expressions of inflammatory markers among the 4 groups, except MCP-1 in group II. However, the mRNA expressions of IL-1β and MCP-1 in the aorta were higher in groups I and II than in group III (**Figure S1 in File S 1**).

### Pathologic features of the liver and aorta

**Figure 2:**
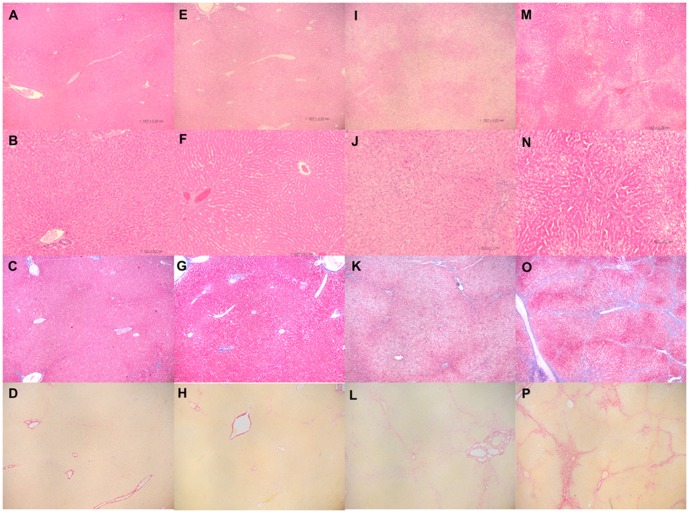
Representative histopathologic features of rabbit liver sections. (A, E, I, M) Hematoxylin–eosin stain, ×40 and (B, F, J, N) ×100′. (C, G, K, O) Masson trichrome stain (×40). (D, H, L, P) Sirius red stain (×40). (A–D) Group I (control) exhibited no fatty changes or fibrosis. (E, F) Liver sections from group II (carrageenan injection) showed no remarkable fatty changes, but mild portal expansion and collagen deposition were observed in sections stained with (G) Masson's trichrome and (H) Sirius red. (I–L) In group III (cholesterol diet), macro- and microvesicular fatty changes were widely observed; the portal areas were widened with sporadic portal–portal fibrosis and portal–central fibrosis. (M–P) Group IV (carrageenan injection and cholesterol diet) exhibited marked macro- and microvesicular fatty changes with frequent portal–portal and portal–central fibrosis. Collagen deposition around fibrotic areas is visualized as red color by Sirius red staining.

There were no pathologic changes in the liver tissue specimens in group I (**Fig. 2A–D**), as fatty changes were primarily inside the hepatocytes. In group II, focal mild expansion of the portal areas with lymphocytic infiltration was observed (**Fig. 2E–H**). Macro- and microvesicular fatty changes were observed in group III but not in group II. Expansion of the portal area due to portal-portal fibrosis and occasional portal-central fibrosis was also observed in group III (**Fig. 2I–L**). Liver specimens from group IV exhibited marked fatty changes and fibrosis (**Fig. 2M–P**). Fibrosis and collagen deposition were easily identified by observing Masson trichrome and Sirius red stained-samples under a low-power microscopic lens (**Fig. 2O, P**). The numbers of portal inflammatory cells were comparable between groups III and IV (**Figures S2, S3 in File S1**). Septal fibrosis with frequent portal-portal and portal-central fibrosis was also present in the liver specimens from group IV (**Figures S2, S3 in File S1**).

**Figure 3:**
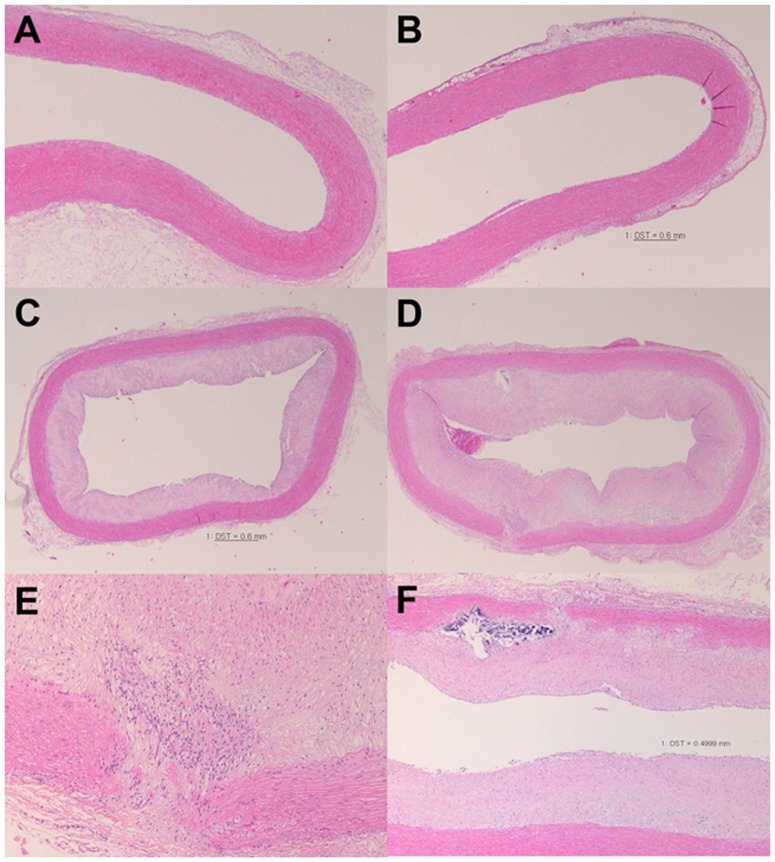
Hematoxylin & eosin-stained sections of rabbit descending aorta. (A) The large lumen of the descending aorta in group I (controls) exhibited a smooth contour with an intact intima and media. (B) Group II (carrageenan injection) showed no remarkable changes in the aortic wall. (C) Atheromatous plaque deposition was evident in group III (cholesterol diet); numerous foam cells without disruption of the internal elastic layer can also be observed. (D) Group IV (carrageenan injection and cholesterol diet) exhibited marked luminal narrowing with advanced atheromatous plaque formation; disruption of the medial layer (left lower portion) and calcification (left upper portion) are also visible. (E) Magnified view of the medial layer disruption showing several foam cells and cholesterol clefts in the breaks within the media. (F) Tissue sample from a group IV rabbit also revealed advanced atheromatous plaques with calcification and medial layer disruption. Original magnification: (C, D) ×40, (A, F) ×100, (B, E) ×200.

Regarding aorta specimens, no intimal or medial pathologic changes were observed in groups I or II (**Fig. 3A, B**). Atherosclerotic plaques of intimal hyperplasia with foam cells were observed in groups III and IV (**Fig. 3C, D** **and Figures S4, S5 in File S1**), but advanced atheromatous plaques with acellular lipid cores were only present in group IV (**Fig. 3D, E** **and Figure S5 in File S1**). Four samples from group IV exhibited medial layer disruption with atheromatous plaques (**Fig. 3D, E** **and Figure S5 in File S1**), which were not observed in group III. Calcification of plaques and/or the medial layer was also evident in group IV (**Fig. 3D, F** **, and Figure S5 in File S1**). The severity of changes in the liver was strongly correlated with that in the descending aorta.

### Cholesterol levels in serum, the liver, and the aorta

Cholesterol accumulation was estimated on the basis of levels of total cholesterol, 2 CEs, and 6 OHCs as oxidative metabolites. The levels of total cholesterol, CEs, and OHCs in serum, the liver, and the aorta were markedly elevated in groups III and IV; they were generally not affected by inflammation, except for the levels of cholesteryl laurate, cholesteryl myristate, and 4β-OHC in serum and the liver (**Table 4**).

**Table 4:** Cholesterol levels in serum, the liver, and the aorta.

Specimen	Compound	Concentration				*p-value*				
		I	II	III	IV	I–II	I–III	I–IV	II–IV	III–IV
Serum	Cholesterol	76.2±54.8	42.1±11.8	2219.7±591.1	1391.9±728.9	NS^a^	0.005	0.015	0.014	NS
	Cholesteryl laurate	0.5	0.2 ± 0.1	ND^b^	3.7±1.4	NC^c^	NC	NC	0.005	NC
	Cholesteryl myristate	11.5±13.2	4.9±1.0	73.0±15.6	164.3±71.8	NS	0.001	0.008	0.008	0.045
	7α-Hydroxycholesterol	24.6±2.6	25.1±0.9	403.1±341.3	272.7±88.8	NS	NS	0.003	0.003	NS
	7β-Hydroxycholesterol	114.9±13.1	130.9±24.0	870.1±288.4	778.1±231.5	NS	0.013	0.003	0.003	NS
	4β-Hydroxycholesterol	61.5±34.4	82.5±18.3	1473.1±559.3	609.8±306.6	NS	0.015	0.016	0.018	0.045
	27-Hydroxycholesterol	ND	ND	ND	ND	NC	NC	NC	NC	NC
	24S-Hydroxycholesterol	ND	ND	ND	ND	NC	NC	NC	NC	NC
	25-Hydroxycholesterol	ND	ND	46.0±21.7	27.0±5.3	NC	NC	NC	NC	NS
Liver	Cholesterol	903.2±54.2	888.9±31.6	2826.8±587.9	2710.5±94.2	NS	0.007	0.000001	0.00001	NS
	Cholesteryl laurate	0.4±0.1	0.3±0.0	1.5±0.2	3.5±1.4	NS	0.001	0.02	0.02	0.058
	Cholesteryl myristate	4.9±1.8	4.3±0.4	67.6±12.7	105.2±43.4	NS	0.002	0.019	0.019	NS
	7α-Hydroxycholesterol	94.2±12.7	98.0±41.4	425.2±209.9	326.9±99.7	NS	0.051	0.018	0.014	NS
	7β-Hydroxycholesterol	1800.2±786.6	1471.9±440.6	3938.9±1926.7	3958.1±831.7	NS	NS	0.007	0.005	NS
	4β-Hydroxycholesterol	1597.9±478.7	1992.4±531.0	6561.2±1735.8	4395.3±946.7	NS	0.008	0.005	0.008	0.084
	27-Hydroxycholesterol	ND	24.6±21.3	176.1±23.7	164.6±84.0	NC	NC	NC	NS	NS
	24S-Hydroxycholesterol	28.4±16.0	24.2±11.3	241.7±76.7	139.7±25.9	NS	0.01	0.001	0.001	0.071
	25-Hydroxycholesterol	ND	ND	ND	ND	NC	NC	NC	NC	NC
Aorta	Cholesterol	1039.0±177.6	986.0±115.6	4308.1±1232.6	7472.9±2459.5	NS	0.042	0.045	0.045	NS
	Cholesteryl laurate	0.4±0.0	0.6±0.2	1.2±0.2	1.5±0.5	0.085	0.015	0.054	0.068	NS
	Cholesteryl myristate	4.6±0.1	4.9±0.2	37.6±16.2	39.8±21.4	0.024	0.072	NS	NS	NS
	7α-Hydroxycholesterol	57.5±25.8	168.8±119.2	433.8±243.5	435.8±161.5	NS	NS	0.053	0.081	NS
	7β-Hydroxycholesterol	196.3±37.0	942.3±115.3	1674.5±998.6	5057.3±3387.3	0.0004	NS	NS	NS	NS
	4β-Hydroxycholesterol	381.1±74.3	498.6±175.8	2636.1±1000.5	2808.6±986.0	NS	0.059	0.05	0.053	NS
	27-Hydroxycholesterol	73.4±17.5	81.6±19.5	366.3±57.8	381.4±194.5	NS	0.009	NS	NS	NS
	24S-Hydroxycholesterol	16.6±4.7	64.6±28.1	115.6±8.2	125.3±37.1	0.04	0.0003	0.035	0.083	NS
	25-Hydroxycholesterol	31.7±9.4	101.4±37.3	409.2±336.7	2441.6±3318.7	0.03	NS	NS	NS	NS

Concentrations of cholesterol, cholesteryl esters, and phytosterols are expressed in µg/mL or µg/g; concentrations of cholesterol precursors and hydroxycholesterols are expressed in ng/mL or ng/g.

I, Group I; II, Group II; III, Group III; IV, Group IV; I–II, Group I versus Group II; I–III, Group I versus Group III; I–IV, Group I versus Group IV; II–IIV, Group II versus Group IV; III–IV, Group III versus Group IV; NS^a^, not significant; ND^b^, not detected; NC^c^, not comparable; CGN, carrageenan; CHO, cholesterol; CON, control.

### Serum lipoprotein electrophoresis

**Figure 4:**
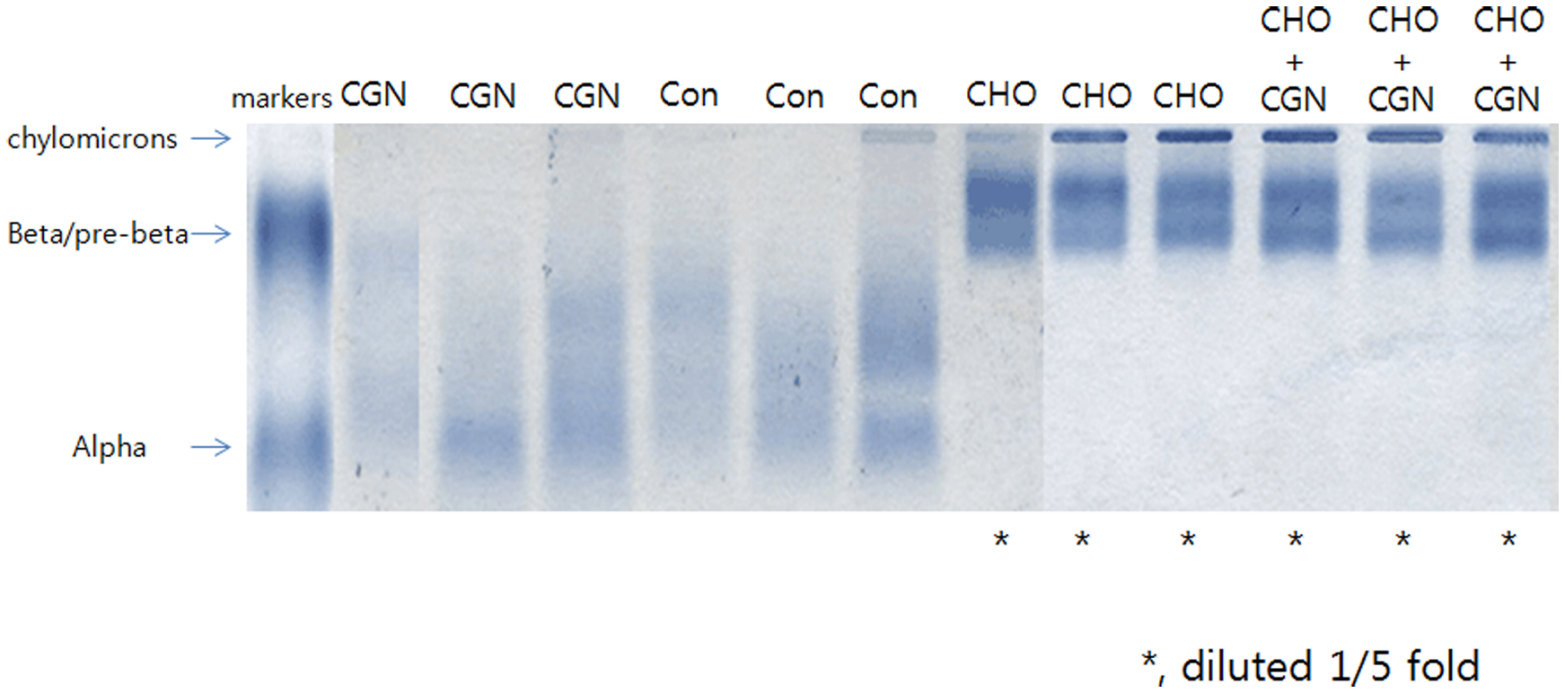
Examples of lipoprotein electrophoresis gels from each group. Groups I (control) and II (carrageenan injection) exhibited normal patterns, and groups III (cholesterol diet) and IV (carrageenan injection and cholesterol diet) exhibited type IV phenotype according to the Fredrickson-Levy-Lees classification. The result shows that inflammation did not affect the lipoprotein phenotype in blood.

Regarding the Fredrickson-Levy-Lees classification, groups I and group II exhibited normal patterns, while groups III and group IV exhibited type IV phenotype. These results indicate inflammation did not affect the lipoprotein phenotype in blood (**Figure 4**).

## Discussion

In this study, rabbits fed a high-cholesterol diet developed early lesions of NAFLD and atherosclerosis of the aorta. Moreover, additional subcutaneous inflammation induced systemic inflammation and accelerated the pathogenesis of lipid-induced damage, leading to advanced lesions in both the liver and aorta. Thus, a two-hit process increased the cholesterol burden in both the liver and arteries, similar to that observed in triglyceride overload. However, high levels of circulatory inflammatory cytokines did not stimulate the development of NAFLD or atherosclerosis in the absence of an overabundance of cholesterol.

Many recent clinical studies have showed that NAFLD is an independent risk factor for CVD in addition to traditional cardiovascular risk factors [21]–[24]. To date, these studies have primarily focused on triglyceride toxicity in the liver rather than cholesterol accumulation. As cholesterol overload results in deposition in the liver, hepatic cholesterol burden is likely to progress to NAFLD, similar to that observed in cases of hepatic triglyceride overload [12], [15], [25], [26]. Lipids and cholesterol can accumulate intracellularly in both the liver and arteries; however, while the presence of cholesterol and triglycerides in the liver results in intracellular deposition in hepatocytes, the arteries respond only to cholesterol via intracellular phagocytosis performed by macrophages derived from the systemic circulation. Cholesterol accumulation in both the liver and arteries augments systemic inflammation in patients with hypercholesterolemia [14]. In the present study, the high-cholesterol diet not only increased proinflammatory cytokine and CRP levels in the blood, but also resulted in the development of early pathologic changes of NAFLD and atherosclerosis. Both transcriptomic and metabolomic analyses demonstrate that a high-cholesterol diet induces a marked systemic inflammatory response in apolipoprotein E3-Leiden transgenic mice [14].

Here, serologic markers of inflammation were similarly elevated in animals fed the high-cholesterol diet alone with or without inflammation induced by carrageenan injection. Despite a lack of differences in the levels of serologic inflammatory markers, both NAFLD and atherosclerosis were highly advanced in the presence of additional inflammation. The finding that TLR expression in the atherosclerotic aorta was more sensitive to inflammatory status provides a plausible explanation as to why atherosclerosis is exacerbated by inflammation. The induction of TLR signaling by intravenous injection of lipopolysaccharides is also reported to increase atherosclerotic plaque volume in hypercholesterolemic rabbits [27]. Although the mechanism by which NAFLD is exacerbated by systemic inflammation is not yet understood, increases in CEs in serum or the liver but not the aorta may be responsible for this vulnerability.

As additional subcutaneous inflammation simultaneously exacerbated existing early lesions caused by cholesterol overload in both the liver and aorta in this study, we examined the cellular responses of inflammatory receptors and cholesterol metabolites in the liver and aorta in order to investigate the mechanism by which the existing lesions were aggravated. The aorta exhibited intimal hyperplasia, foam cells, and elevated expression mRNA of TLR-2 and TLR-4 in diet-induced hypercholesterolemia. In contrast, systemic inflammation induced by the regular subcutaneous injection of carrageenan (a TLR-4 ligand) minimally increased aortic mRNA expression of TLR-2 and TLR-4 without any atherosclerotic lesions in normocholesterolemic rabbits. The addition of systemic inflammation to hypercholesterolemia significantly increased mRNA expression of TLR-2 and TLR-4 and exacerbated atherosclerotic lesions within the aorta. Accordingly, mRNA expression of TLR-2 and TLR-4 in the aorta is associated with the severity of atherosclerosis and serum levels of inflammatory markers in hypercholesterolemic rabbits. However, it is impossible to know which cells had elevated expressions of these TLRs from the results of this study, even though it seems likely that the increased TLR expression in the aorta was mainly from macrophages. Moreover, the present results do not clarify the mechanism of TLR-2 expression. The expressions of TLR-2 and TLR-4 are elevated in animal and human atherosclerotic lesions [28], [29]. In addition, TLR-4 and to a lesser extent TLR-2 contribute to early-stage intimal foam cell accumulation at lesion-prone aortic sites in ApoE-deficient mice [30]. TLR-4 signaling upregulates TLR-2 gene expression in endothelial cells, smooth muscle cells, and macrophages both in vitro [31]–[33] and in vivo in ApoE-deficient mice [30]. Endothelial cell-specific TLR-2 expression is also upregulated via shear stress and hypercholesterolemia independent of TLR-4 expression [34]. Therefore, the TLR-2 expression observed in this study may be related to TLR-4 upregulation and/or hypercholesterolemia. In contrast, mRNA expression of TLR-2 and TLR-4 in the liver was quite different from that observed in the aorta. The expressions of these TLRs were slightly elevated in the presence of cholesterol and/or inflammation regardless of treatment modality or liver pathology. This result is consistent with the results of a previous study showing that the in vivo responses of both hepatocytes and Kupffer cells to TLR-2 and TLR-4 ligands are very weak [35]. Regarding other inflammatory genes, the mRNA expressions of IL-β, TNF-α, and MCP-1 in the liver did not differ significantly among the 4 experimental groups, except for MCP-1 in group II. However, the mRNA expressions of IL-1β and MCP-1 in the aorta were elevated in group IV. These findings contrast with those of TLR expression; therefore, further studies are required to confirm their underlying mechanisms.

We also evaluated the amounts of cholesterol, CEs, and OHCs in serum, the liver, and the aorta. Serum cholesterol levels were significantly elevated in rabbits fed the cholesterol diet (**Table 4**). Although total cholesterol, free cholesterol, and OHCs tended to decrease with additional inflammatory load in group IV, the levels of 2 CEs (cholesteryl laurate and myristate) increased in both serum and the liver. CEs are formed within the circulation by lecithin-cholesterol acyltransferase, an enzyme produced by the liver. The blood is carried into the liver via anchor lipid molecules, and it subsequently enters the systemic circulation to supply metabolic energy. Although NAFLD is characterized by the accumulation of both triglycerides and free cholesterol without a corresponding increase in CEs [36], elevated CE levels in the presence of systemic inflammation may play a biochemical role in cholesterol accumulation in the liver. This could also be exacerbated by inflammation due to unrelated inflammatory disease, although further studies are required to investigate the underlying mechanism. This study revealed that serum levels of lipids including total cholesterol, triglycerides, and LDL-C, albumin as well as WBC counts are slightly lower in rabbits that received the cholesterol diet plus inflammatory stimulus (group IV) than those fed the cholesterol diet only (group III). OHCs are generally converted into acidic products because of slow metabolism, and the accumulation of oxidized cholesterols in advanced atherosclerotic lesions contributes significantly to plaque vulnerability [37]. In particular, 4β-OHC has an unusually long half-life in the blood, but inflammatory stress can exacerbate lipid accumulation in the hepatic cells and fatty livers of ApoE-deficient mice [13] by increasing the expressions of LDL receptor and sterol regulatory genes for cholesterol synthesis and inhibiting the expressions of genes responsible for cholesterol efflux. However, the addition of inflammation did not alter aortic cholesterol metabolites in the present study. This may also be related to the decreased levels of cholesterol metabolites in blood and the liver with the deterioration of liver function because of inflammation. The cholesterol diet induced Fredrickson-Levy-Lees phenotype type IV hyperlipidemia in this rabbit model, and inflammation did not affect the lipoprotein phenotype in the blood.

The aorta and liver handled cholesterols differently, which may reflect the presence of specific receptor proteins and pathways for cholesterol metabolism. Regardless of the differences in the mechanisms of cholesterol accumulation and responses to inflammation in these 2 tissues, the impact of tissue damage due to remote inflammation can be considerable in both the liver and aorta. The deposition of triglycerides is suggested to increase vulnerability to further injury in NAFLD caused by triglyceride accumulation. However, it remains unclear how remote systemic inflammation exacerbates NAFLD and atherosclerosis. Clinical studies indicate a high prevalence of CVD in patients with systemic inflammatory diseases [38]–[40] as well as an increased frequency of liver abnormalities in certain chronic inflammatory conditions [41], [42]. This study used regular subcutaneous injections of TLR-4 ligand (i.e., carrageenan) to induce systemic inflammation in rabbits. The results demonstrate that remote subcutaneous inflammation resulted in the aggravation of atherosclerotic lesions in the aorta, progression of inflammation and fibrosis of NAFLD in the liver, and elevated expressions of serologic markers in blood. In group IV, the expressions of TLR-2 and TLR-4 as well as some inflammatory cytokines were elevated in the aorta, while CE levels were significantly elevated in the liver but not the aorta. Nevertheless, these changes are insufficient to fully explain the observed changes in the lesions caused by inflammation in this model. Therefore, further precise studies are required.

This study has several limitations. First, the experiments were conducted in rabbits, which have a lipid metabolism different from that of humans. In addition, rabbits fed the cholesterol diet had very high serum levels because of an inability to increase sterol excretion [43], [44]. Only a few relevant fundamental studies have been performed on animals or humans. Second, we examined fat accumulation in the liver due to cholesterol rather than triglycerides, and observed pathologic findings typical of those observed in NAFLD. It is noteworthy that cholesterol can induce the same pathology as NAFLD without the typical lipotoxicity of triglycerides, because cholesterol, CEs, and OHCs can accumulate in arteries in addition to the liver. Accordingly, we measured cholesterol, CEs, and OHCs in the aorta instead of plaques. To the best of our knowledge, this is the first study evaluating individual cholesterol levels with respect to underlying atherosclerosis and/or NAFLD. Finally, we used subcutaneous injection to induce local and systemic inflammation because of the higher toxicity of lipopolysaccharides (pure ligands of TLR-4), which induced liver cirrhosis when administered for 3 months. Carrageenan also acts as a ligand of TLR-4 but has some additional biological effects [45]–[46]. Despite these limitations, it is widely used in animal studies as a reactant by either subcutaneous or intrapleural administration to induce non-specific inflammatory reactions, which may be a consequence of the activation of innate immunity via pattern recognition receptors. Carrageenan administration increased WBC counts and inflammatory marker levels in the blood.

In conclusion, dietary cholesterol overload induced the development of early lesions of NAFLD in the liver and atherosclerosis in the aorta of rabbits. Additional subcutaneous inflammation in diet-induced hypercholesterolemic rabbits accelerated the pathologic process, resulting in advanced lesions in both organs. Thus, high cholesterol burden acts via a two-hit process similar to that observed in triglyceride overload, resulting in both liver and arterial damage. Systemic inflammation induced by local injection of carrageenan, a TLR-4 ligand, did not damage the liver or aorta in the absence of cellular cholesterol accumulation. Therefore, the role of inflammation in the progression of disease may depend on the presence of underlying pathology in both tissues.

## Supporting Information

File S1 **Table S1 and Figures S1–S5. Table S1.** PCR primer sequences and conditions used in this study. **Figure S1.** In the hepatic tissue, there was no difference of mRNA expression of inflammatory genes among the 4 experimental groups except MCP-1 in CGN group. However, there were higher mRNA expression of IL-1β and MCP-1 of aorta in cholesterol fed and CGN injected group. T bars indicate one standard deviation. **Figure S2.** Dot plot figures of histologic analysis of rabbit liver tissues. Left figure shows steatosis (%) of each sample. Steatosis of group III and group IV samples were significantly higher than group I and group II samples (group I, II VS group III, *p* = 0.031; group I, II VS group IV, *p* = 0.004). Difference of fatty change between group III and group IV samples did not show statistical significance (group III VS group IV, *p* = 0.090). Middle figure shows NAS (NAFLD activity score) of each sample. NAS scores of group III and group IV rabbits were significantly higher than group I and group II rabbits (*p*<0.001). NAS score of group IV rabbits was also higher than group III rabbits (*p* = 0.043). Right figure demonstrates fibrosis scores of each sample. Liver fibrosis was more evidently observed in group III and group IV rabbits compared to group I and group II rabbits (*p*<0.001). Fibrosis of group IV rabbits was higher than group III rabbits, but statistical significance was marginal (*p* = 0.111). **Figure S3.** Microscopic figures of liver tissues from rabbits. Portal areas (A, C, E and G) and central areas (B, D, F and H) are magnified (Hematoxylin and eosin, original magnification x200). No steastosis and fibrosis was observed in group I (A, B) and group II (C, D) samples. Group III (E, F) samples show pericentral steatosis, and group IV (G, H) samples reveal pericentral steatosis and pericellular fibrosis. Mild infiltration of inflammatory cells mainly composed of lymphocytes were observed in portal areas of group II, III and IV rabbits, but the difference between these groups were not observed. **Figure S4.** Representative microscopic figures of aorta from the Group III rabbits. (Hematoxylin and eosin; original magnification x40). Group III samples show earlier lesions composed of thick intimal hyperplasia, but acellular lipid core or fibrous cap was not observed in any animal of this group. **Figure S5.** Representative microscopic figures of aorta from the Group IV rabbits. (A–F, Hematoxylin and eosin; original magnification x40, G–H, Hematoxylin and eosin; original magnification x 400). Five of 6 animals had typical advanced atherosclerotic lesions with necrotic core and/or scattered calcifications. Four animals have medial interruption with leukocyte infiltration in atheromatous plaque(arrows of D and E; G and H with magnification).
(DOC)
