# Supplementary material for: Cholesterol-Induced Non-Alcoholic Fatty Liver Disease and Atherosclerosis Aggravated by Systemic Inflammation
Source: PLoS One. 2014 Jun 5;9(6):e97841. doi: 10.1371/journal.pone.0097841 (PMC4046981; doi:10.1371/journal.pone.0097841)
Supplement: File S1 — Table S1 and Figures S1–S5. Table S1. PCR primer sequences and conditions used in this study. Figure S1. In the hepatic tissue, there was no difference of mRNA expression of inflammatory genes among the 4 experimental groups except MCP-1 in CGN group. However, there were higher mRNA expression of IL-1β and MCP-1 of aorta in cholesterol fed and CGN injected group. T bars indicate one standard deviation. Figure S2. Dot plot figures of histologic analysis of rabbit liver tissues. Left figure shows steatosis (%) of each sample. Steatosis of group III and group IV samples were significantly higher than group I and group II samples (group I, II VS group III, p = 0.031; group I, II VS group IV, p = 0.004). Difference of fatty change between group III and group IV samples did not show statistical significance (group III VS group IV, p = 0.090). Middle figure shows NAS (NAFLD activity score) of each sample. NAS scores of group III and group IV rabbits were significantly higher than group I and group II rabbits (p<0.001). NAS score of group IV rabbits was also higher than group III rabbits (p = 0.043). Right figure demonstrates fibrosis scores of each sample. Liver fibrosis was more evidently observed in group III and group IV rabbits compared to group I and group II rabbits (p<0.001). Fibrosis of group IV rabbits was higher than group III rabbits, but statistical significance was marginal (p = 0.111). Figure S3. Microscopic figures of liver tissues from rabbits. Portal areas (A, C, E and G) and central areas (B, D, F and H) are magnified (Hematoxylin and eosin, original magnification x200). No steastosis and fibrosis was observed in group I (A, B) and group II (C, D) samples. Group III (E, F) samples show pericentral steatosis, and group IV (G, H) samples reveal pericentral steatosis and pericellular fibrosis. Mild infiltration of inflammatory cells mainly composed of lymphocytes were observed in portal areas of group II, III and IV rabbits, but the difference between these [file pone.0097841.s001.doc]

***Supplementary Information for***

Cholesterol-induced non-alcoholic fatty liver disease and atherosclerosis aggravated by systemic inflammation

**Eung Ju Kim^*,1^, Baek-hui Kim^†,1^, Hong Seog Seo^*‡,2^ Man Ho Choi^&,2^, Yong Jik Lee*, Hyun Hee Kim^*^, Hyun-Hwa Son^&^**

^*^Cardiovascular Center, Division of Cardiology, Department of Internal Medicine and **^†^**Department of Pathology, Korea University Guro Hospital, Korea University College of Medicine, Seoul 152-703, Korea. **^‡^** the Korea University-Korea Institute of Science and Technology (KU-KIST) Graduate School of Converging Science and Technology. **^&^**Future Convergence Research Division, Korea Institute of Science and Technology, Seoul 136-791, Korea

^1,2^These authors contributed equally to this work as a first author and corresponding author.

**Table S1. PCR primer sequences and conditions used in this study**

| **gene name** | **primer sequence** | **Tm** | **product size**  **(base pairs)** |
| --- | --- | --- | --- |
| **Rabbit MCP-1** | **LEFT PRIMER gaattccccagtcacctgct**  **RIGHT PRIMER tttgggacacttggtgctgt** | **52 ℃** | **100** |
| **Rabbit IL1β** | **LEFT PRIMER gctttgggattctctccagc**  **RIGHT PRIMER cttctccagagccacaacga** | **54 ℃** | **271** |
| **Rabbit GAPDH** | **LEFT PRIMER agaacatcatccctgcctcc**  **RIGHT PRIMER ttgaagtcgcaggagacgac** | **54 ℃** | **250** |
| **Rabbit TNF** | **LEFT PRIMER atgaagctcacggacaacca**  **RIGHT PRIMER gaggttgaccttgttcgggt** | **52 ℃** | **156** |

**Figure S1**


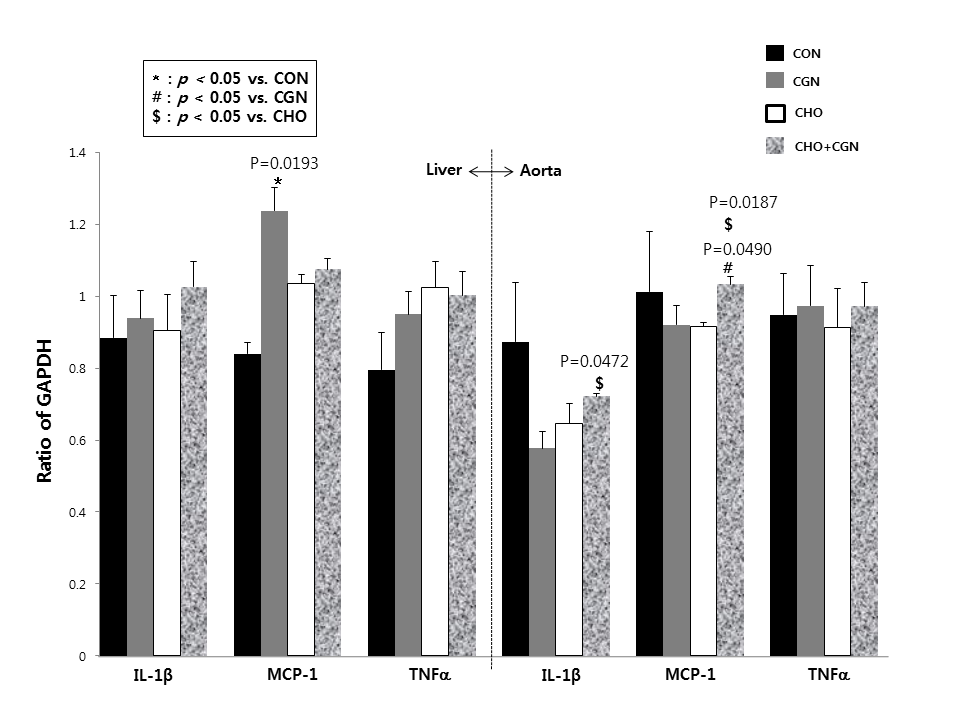


Figure S1. In the hepatic tissue, there was no difference of mRNA expression of inflammatory genes among the 4 experimental groups except MCP-1 in CGN group. However, there were higher mRNA expression of IL-1β and MCP-1 of aorta in cholesterol fed and CGN injected group. T bars indicate one standard deviation

**Figure S2**


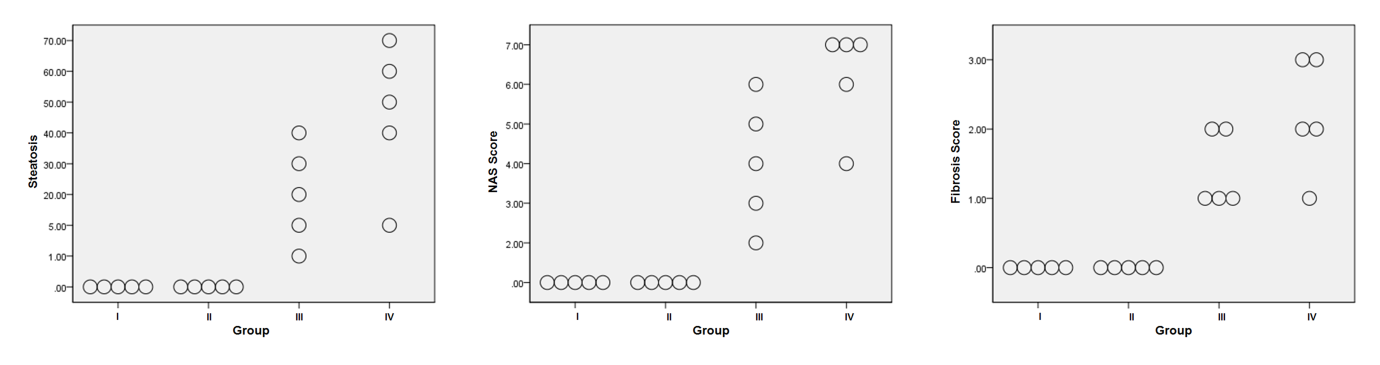


Figure S2. Dot plot figures of histologic analysis of rabbit liver tissues. Left figure shows steatosis (%) of each sample. Steatosis of group III and group IV samples were significantly higher than group I and group II samples (group I, II VS group III, *p*=0.031; group I, II VS group IV, *p*=0.004). Difference of fatty change between group III and group IV samples did not show statistical significance (group III VS group IV, *p*=0.090). Middle figure shows NAS (NAFLD activity score) of each sample. NAS scores of group III and group IV rabbits were significantly higher than group I and group II rabbits (*p*<0.001). NAS score of group IV rabbits was also higher than group III rabbits (*p*=0.043). Right figure demonstrates fibrosis scores of each sample. Liver fibrosis was more evidently observed in group III and group IV rabbits compared to group I and group II rabbits (*p*<0.001). Fibrosis of group IV rabbits was higher than group III rabbits, but statistical significance was marginal (*p*=0.111).

**Figure S3**


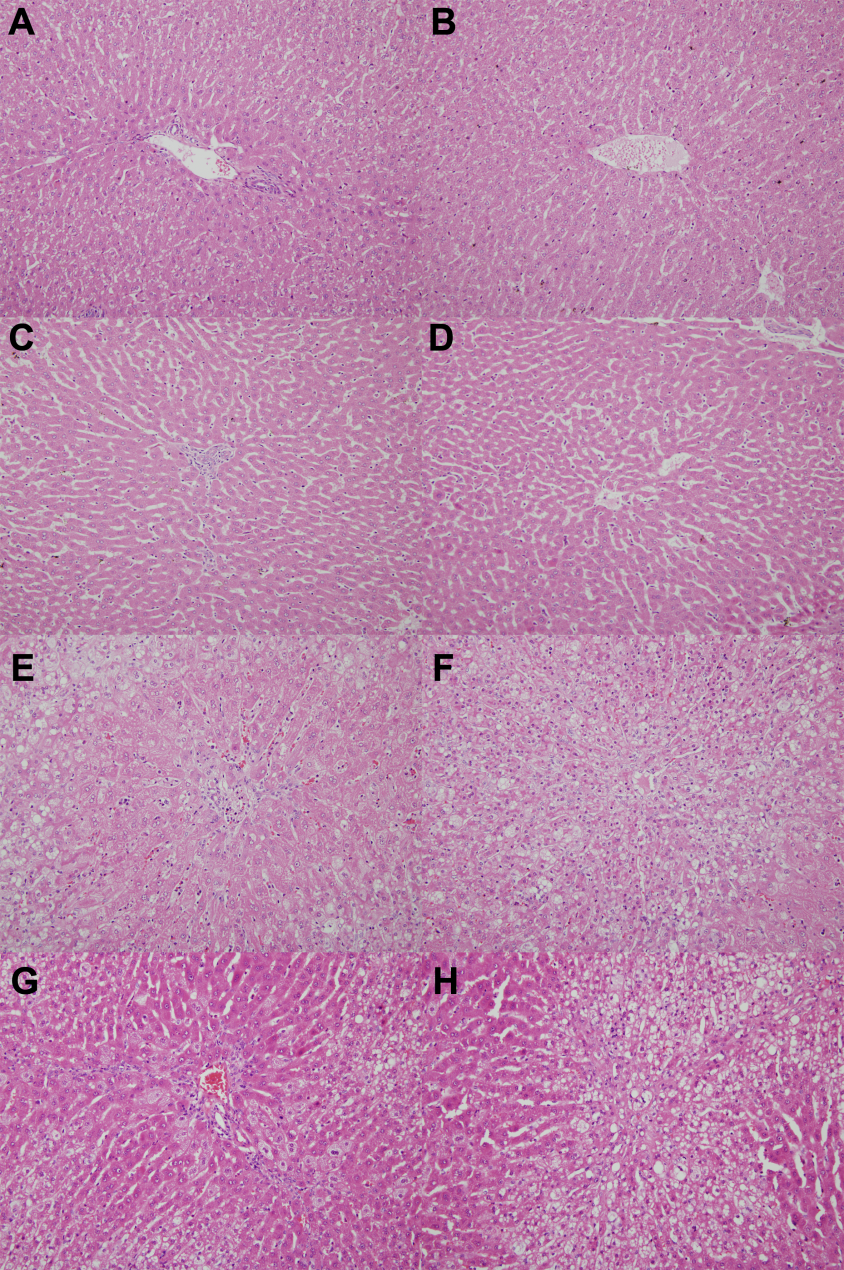


Figure S3. Microscopic figures of liver tissues from rabbits. Portal areas (A, C, E and G) and central areas (B, D, F and H) are magnified (Hematoxylin and eosin, original magnification x200). No steastosis and fibrosis was observed in group I (A, B) and group II (C, D) samples. Group III (E, F) samples show pericentral steatosis, and group IV (G, H) samples reveal pericentral steatosis and pericellular fibrosis. Mild infiltration of inflammatory cells mainly composed of lymphocytes were observed in portal areas of group II, III and IV rabbits, but the difference between these groups were not observed.

**Figure S4**


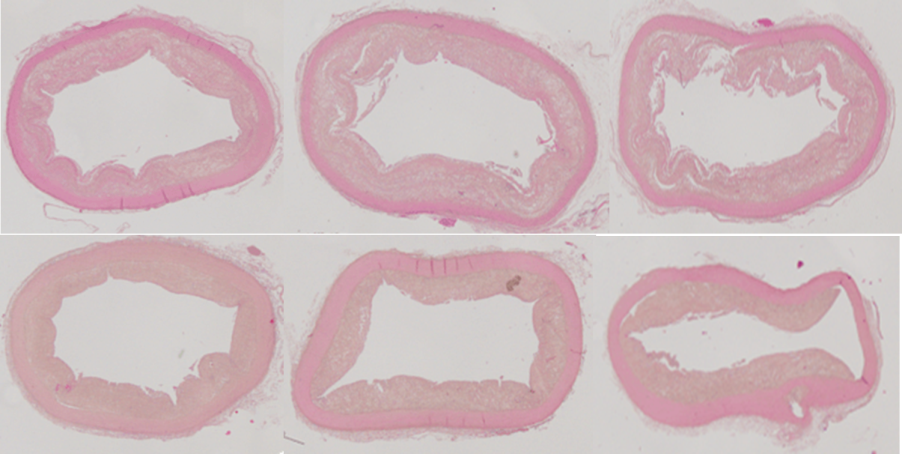


Figure S4. Representative microscopic figures of aorta from the Group III rabbits. (Hematoxylin and eosin; original magnification x40). Group III samples show earlier lesions composed of thick intimal hyperplasia, but acellular lipid core or fibrous cap was not observed in any animal of this group.

**Figure S5**


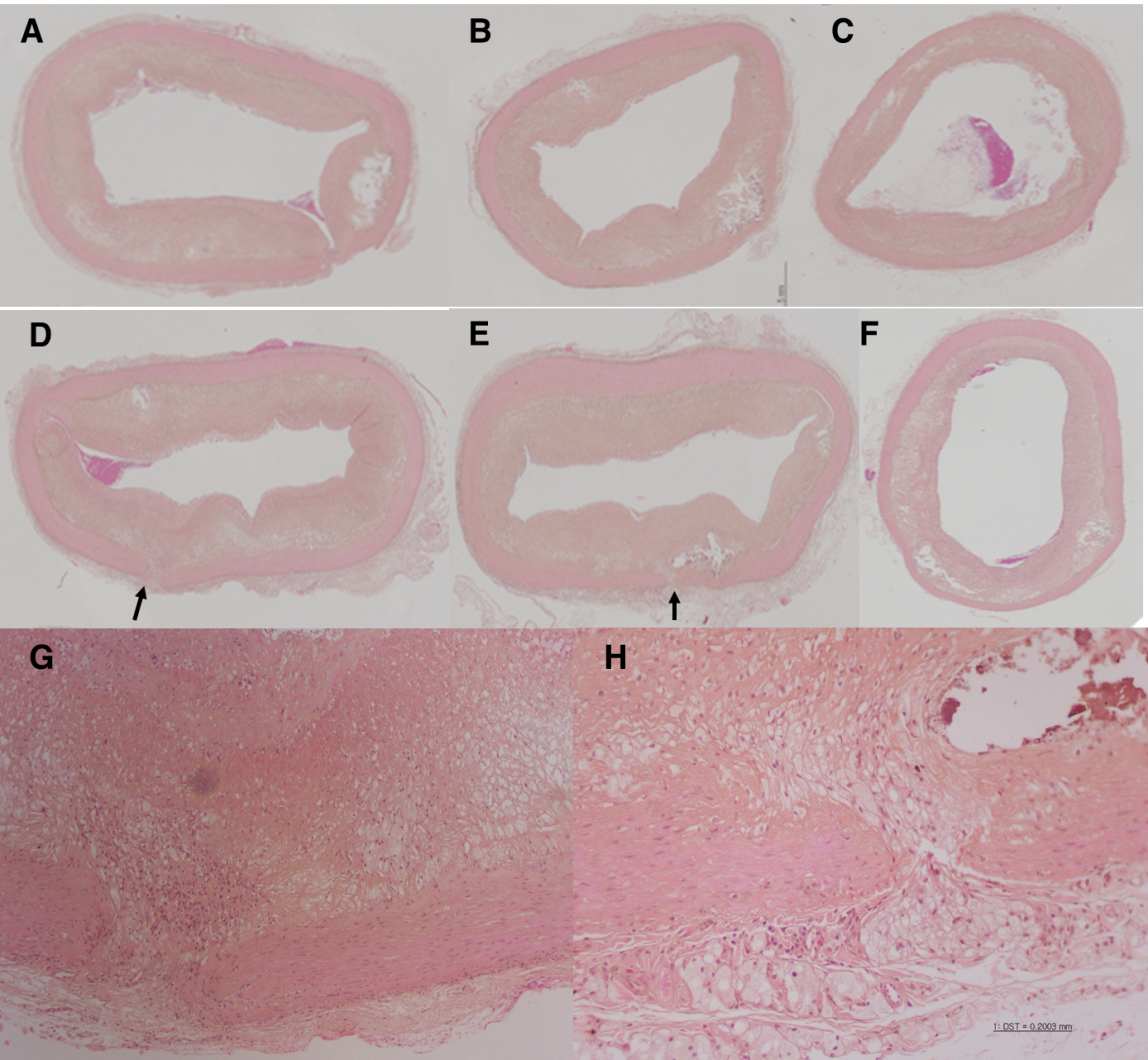


Figure S5. Representative microscopic figures of aorta from the Group IV rabbits. (A-F, Hematoxylin and eosin; original magnification x40, G-H, Hematoxylin and eosin; original magnification x 400). Five of 6 animals had typical advanced atherosclerotic lesions with necrotic core and/or scattered calcifications. Four animals have medial interruption with leukocyte infiltration in atheromatous plaque(arrows of D and E; G and H with magnification).
